# Association Between Delayed/Forgone Medical Care and Resource Utilization Among Women with Breast Cancer in the United States

**DOI:** 10.1245/s10434-024-16586-x

**Published:** 2024-12-18

**Authors:** Kriyana P. Reddy, Kathleen Jarrell, Cara Berkowitz, Sarah Hulse, Leisha C. Elmore, Rebecca Fishman, Rachel A. Greenup, Alina M. Mateo, Jami D. Rothman, Dahlia M. Sataloff, Julia C. Tchou, S. Yousuf Zafar, Oluwadamilola M. Fayanju

**Affiliations:** 1 Division of Breast Surgery, Department of Surgery, Perelman School of Medicine, The University of Pennsylvania, Philadelphia, PA USA; 2 Department of Surgery, Thomas Jefferson University Hospital, Philadelphia, PA USA; 3 Department of Surgery, Weill Cornell School of Medicine, New York, NY USA; 4 Department of Surgery, Yale University School of Medicine, New Haven, CT USA; 5 Rena Rowan Breast Center, Abramson Cancer Center, The University of Pennsylvania, Philadelphia, PA USA; 6 Leonard Davis Institute of Health Economics, The University of Pennsylvania, Philadelphia, PA USA; 7 Department of Medicine, Duke University School of Medicine, Durham, NC USA; 8 Penn Center for Cancer Care Innovation, Abramson Cancer Center, The University of Pennsylvania, Philadelphia, PA USA

## Abstract

**Background:**

Although high treatment costs of breast cancer care are well documented, the relationship between delayed/forgone (D/F) care and resource utilization among patients with breast cancer is unknown. This study sought to investigate the relationship between D/F care, resource use, and healthcare expenditures among patients with breast cancer.

**Methods:**

Data on adult female patients with breast cancer were obtained from the Medical Expenditure Panel Survey to assess resource utilization and expenditures in the United States from 2007 to 2017. Weighted proportions of patients with ≥ 1 emergency department, ≥ 1 inpatient, ≥ 1 outpatient, and > 5 office-based encounters were compared between those experiencing D/F care versus those who did not using Rao-Scott adjusted chi-squared tests. Annual, per capita total, out-of-pocket, emergency department, inpatient, outpatient, office-based visit, and prescription medication expenditures were compared by using two-part econometric models.

**Results:**

Five percent of patients with breast cancer experienced D/F care, and 42.9% of patients cited financial barriers as the primary reason for D/F care. In unweighted estimates, there were higher proportions of patients with ≥ 1 hospitalizations (37% vs. 16%, *P* < 0.001) among those experiencing D/F care versus those who did not. Patients with D/F care had $5372 (95% CI $35–$10,709, *P* = 0.04) higher per capita inpatient expenditures than patients without D/F care.

**Conclusions:**

Delayed/forgone care is associated with increased resource utilization and healthcare spending among breast cancer patients. Further work is needed to address the root causes of D/F breast cancer care, with a view to mitigating disparate outcomes and increasing costs.

**Supplementary Information:**

The online version contains supplementary material available at 10.1245/s10434-024-16586-x.

Breast cancer is the most common form of cancer and the second most common cause of death from cancer among women worldwide.^1,2^ Furthermore, breast cancer has the highest treatment costs of any cancer in the United States, accounting for 14% of the U.S. total.^3^ These costs are projected to increase. An aging population, increasing costs of medical treatment and screening, and changes in incidence and survival within population subgroups have been cited as reasons for burgeoning costs.^4,5^ Notably, the share of cancer treatments that are paid by Medicare is increasing, and a projected 73% of cancer survivors will be 65 years or older by 2040.^6^ In one study, Medicare payments were three times greater than private insurance payments among older-aged breast cancer patients.^7^ Thus, the growing financial burden of cancer care not only on patients but also on taxpayers and public payors remains a significant policy issue.

Timeliness of care may correlate with treatment costs among patients with breast cancer. Treatment costs vary by stage at diagnosis, and advanced stage diagnosis is associated with higher resource utilization and inferior outcomes.^8,9^ The 5-year survival in women receiving surgical intervention for breast cancer is significantly lower for those facing treatment delays compared with those who do not.^10^ Despite the prognostic implications of delayed care, the relationship between delayed and forgone care and healthcare resource utilization remains unknown. In this study, we describe the prevalence of delayed or foregone (D/F) medical care as well as the association between D/F care and healthcare resource utilization and expenditures among women with breast cancer in the United States.

## Methods

This study was deemed exempt by the institutional review board at the University of Pennsylvania. No informed consent was required for this study. The study followed the Consolidated Health Economic Evaluation Reporting Standards (CHEERS).^11^

### Data Source and Study Cohort

The Medical Expenditure Panel Survey (MEPS) is a series of nationally representative cross-sectional surveys that collect information from a sample of U.S. households on the sociodemographic and financial characteristics, medical conditions, health resource use, and associated costs of each household’s members. Respondents are each assigned a person-weight and variance estimation stratum to ensure national representativeness and adjust for unequal probabilities of selection, nonresponse, and other sampling biases. We collected annual full-year consolidated and medical conditions data files from MEPS for years 2007 to 2017. Full-year consolidated data files contain information on patients’ healthcare expenditures, healthcare encounters, sociodemographic characteristics, self-reported D/F necessary medical care, and self-reported reasons for D/F necessary medical care. Medical conditions files provide information on respondents’ medical conditions in the year preceding survey response in the form of International Classification of Diseases, 9th Edition, Clinical Modification (ICD-9-CM) and International Classification of Diseases, 10th Edition, Clinical Modification (ICD-10-CM) codes. Annual files were pooled together to obtain a large enough sample for determining precise estimates.

The study cohort included adult (age ≥ 18 years) females with a diagnosis of breast cancer in the 12 months preceding survey response, which was determined by ICD-9-CM codes 174 and 233 and ICD-10-CM code C50, and nonmissing data on D/F care.

### Outcomes

The primary outcomes were self-reported D/F medical care, healthcare encounters, and healthcare expenditures in the 12-month period preceding the respondents’ completion of MEPS. Each participant was asked whether she had delayed necessary medical care in the preceding year (yes/no) and if she had forgone necessary medical care in the preceding year (yes/no). If a participant answered “yes” to either question, we considered her as having experienced D/F care. If a participant answered “yes” to either question, she was asked a follow-up question as to the reason for their D/F care. Reasons were classified as “financial,” “nonfinancial,” or “other” as detailed in Supplemental Methods 1.

To evaluate each participant’s healthcare encounters, we collected data on the number of emergency department (ED) visits, inpatient hospitalizations, outpatient visits, and office-based visits each participant had in the year preceding survey response. To evaluate each participant’s healthcare expenditures, we identified annual per capita total healthcare expenditures, out-of-pocket expenditures, ED expenditures, inpatient expenditures, outpatient expenditures, office-based visit expenditures, and prescription medication expenditures in the year preceding survey response. All expenditures were inflation-adjusted using values of the gross domestic product deflator obtained from the Organisation of Economic Co-operation and Development and are presented as constant 2017 U.S. dollars.

### Statistical Analysis

Weighted baseline characteristics of the study cohort were summarized as counts (proportions) for categorical variables and medians (IQRs) for continuous variables. Baseline characteristics included age, race and ethnicity (Non-Hispanic [NH] White, NH Black, NH Asian, NH Other or Multiple Races, and Hispanic), family income as % of poverty line (poor or near poor [0–125%], low income [125–199%], middle income [200–399%], and high income [≥ 400%]), insurance type (private, public, and uninsured), educational attainment (less than GED, GED or high school diploma, and some college or more), census region of residence (northeast, midwest, south, west), and Charlson Comorbidity Index. Charlson Comorbidity Index was operationalized as a categorical variable (scores of 1, 2, and ≥ 3). Baseline characteristics among patients experiencing D/F care were compared with characteristics of those not experiencing D/F care using Rao-Scott adjusted chi-squared tests (for proportions) and Kruskal–Wallis tests (for medians).

To determine factors associated with experiencing D/F care, univariate unadjusted and multivariate adjusted logistic regression models were fit. Delayed/forgone care was the dependent variable. Age, race and ethnicity, family income as % of poverty line, insurance type, educational attainment, region, and Charlson Comorbidity Index were the independent variables. All logistic regression models accounted for survey weights and the complex survey design of MEPS.

To compare the frequency of healthcare encounters between those experiencing D/F care versus those who did not, unweighted proportions of patients having ≥ 1 ED visit, ≥ 1 hospitalization, and ≥ 1 outpatient visits were compared using Rao-Scott adjusted chi-squared tests. Because the distribution of office-based visits was not zero-inflated, proportions of respondents with 0–2, 2–5, and > 5 office-based visits were compared using Rao-Scott adjusted chi-squared tests. All proportions were compared first within the overall study cohort and subsequently within the following subgroups: respondents aged ≥ 65 years; respondents aged < 65 years; respondents whose family income was poor or near poor; and respondents whose family income was low, middle, or high.

Data on healthcare expenditures were zero-inflated. Therefore, two-part models were used to compare healthcare expenditures between those experiencing D/F care versus those who did not experience D/F care. The first part is a logit model that determines the probability of nonzero, positive expenditures. The second part is a generalized linear model with gamma distribution and logarithmic link function that estimates average annual per capita expenditures. Two-part models were separately fit to estimate each category of expenditures. The independent variable of interest was a binary variable indicating D/F care. All expenditure models were adjusted for insurance type, because insurance coverage was an independent, significant predictor of expenditures. The *twopm* command in Stata 18.0 was used to fit the two-part models, and average marginal effects were estimated by using the *margins* command. All expenditure models accounted for the complex survey design of MEPS. All models were constructed in Stata version 18.0 (Statacorp, USA). All statistical testing was 2-tailed, with *P* values < 0.05 considered statistically significant.

## Results

**Fig. 1:**
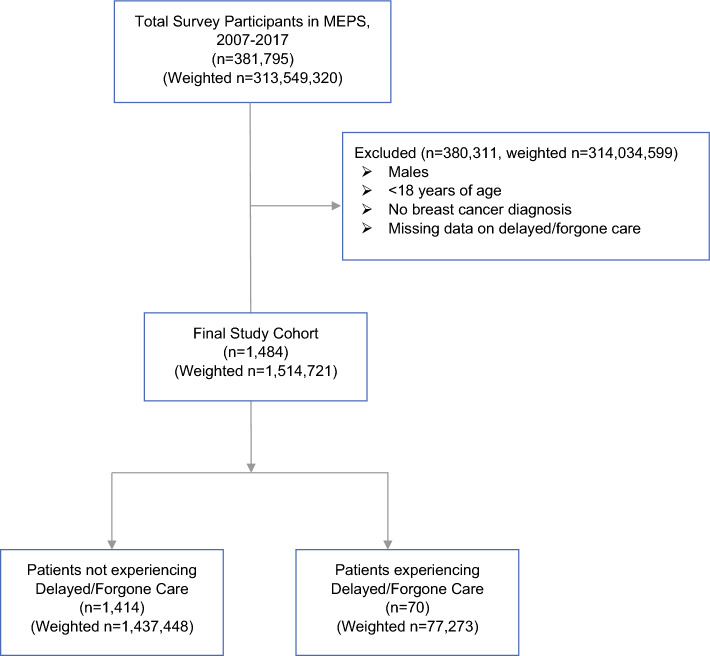
CONSORT diagram of cohort selection: women with breast cancer from MEPS data, 2007 to 2017

In 2007–2017, there were 381,795 MEPS respondents, representing a weighted population of 313,549,320 individuals nationwide. We identified 1484 (weighted n = 1,514,721) adult females with a diagnosis of breast cancer and nonmissing data on D/F care. Among those individuals, an unweighted 4.7% reported experiencing D/F care. The weighted proportion of individuals within the study cohort experiencing D/F care was 5.1% (Fig. 1).

Among patients reporting D/F care, 31.6% were poor or near poor compared with 12.6% of patients not reporting D/F care (*P* = 0.003). A higher proportion of patients experiencing D/F care had ≥ 1 hospitalization than patients who did not report D/F care (36.7% vs. 15.7%, *P* < 0.001) (Table 1).

**Table 1:** Baseline characteristics of women with breast cancer in 2007–2017 MEPS, weighted estimates

	No delayed/forgone care	Delayed/forgone care	*P* value
Sample, n	1414	70	
Weighted sample, n	1,437,448	77,273	
Age, yr, median (IQR)	65 (57 to 75)	61 (53 to 73)	0.83
Race and ethnicity, n (%)			
Non-Hispanic White	1,125,598 (78.3)	57,463 (74.4)	0.46
Non-Hispanic Black	143,536 (9.9)	11,110 (14.4)	
Non-Hispanic Asian	52,037 (3.6)	817 (1.1)	
Non-Hispanic other or multiple races	16,024 (1.1)	1,237 (1.6)	
Hispanic	100,253 (6.9)	106,899 (7.1)	
Family income as % of poverty line, n (%)			
Poor or near poor	181,459 (12.6)	24,453 (31.6)	0.003
Low income	188,757 (13.1)	10,991 (14.2)	
Middle income	401,139 (27.9)	17,959 (23.2)	
High income	666,092 (46.3)	23,969 (30.9)	
Insurance type, n (%)			
Private	1,017,755 (70.8)	42,083 (54.5)	0.09
Public	391,134 (27.2)	30,842 (39.9)	
Uninsured	28,558 (1.9)	4,348 (5.6)	
Educational attainment, n (%)			
Less than GED	131,020 (9.8)	6,331 (10.2)	0.95
GED or HS Diploma	663,823 (49.5)	31,728 (51.2)	
Some college or more	547,052 (40.8)	23,875 (28.5)	
Region, n (%)			
Northeast	311,332 (21.7)	11,014 (14.2)	0.27
Midwest	331,775 (23.1)	13,612 (17.6)	
South	509,197 (35.4)	29,569 (38.4)	
West	285,143 (19.8)	22,987 (29.7)	
Charlson comorbidity index, n (%)			
1	1,079,835 (75.1)	55,513 (71.8)	0.78
2	284,573 (19.8)	18,018 (23.3)	
3+	73,040 (5.1)	3742 (4.8)	
≥ 1 ED visits, n (%)	254,112 (17.7)	18,435 (23.9)	0.30
≥ 1 hospitalizations, n (%)	226,041 (15.7)	28,322 (36.7)	< 0.001
≥ 1 outpatient visits, n (%)	714,440 (49.7)	48,577 (62.9)	0.11
No. office-based visits, n (%)			
0–2 visits	123,327 (8.6)	12,207 (15.8)	0.29
3–5 visits	235,207 (16.4)	7103 (9.2)	
> 5 visits	1,078,914 (75.1)	57,963 (75.0)	

**Fig. 2:**
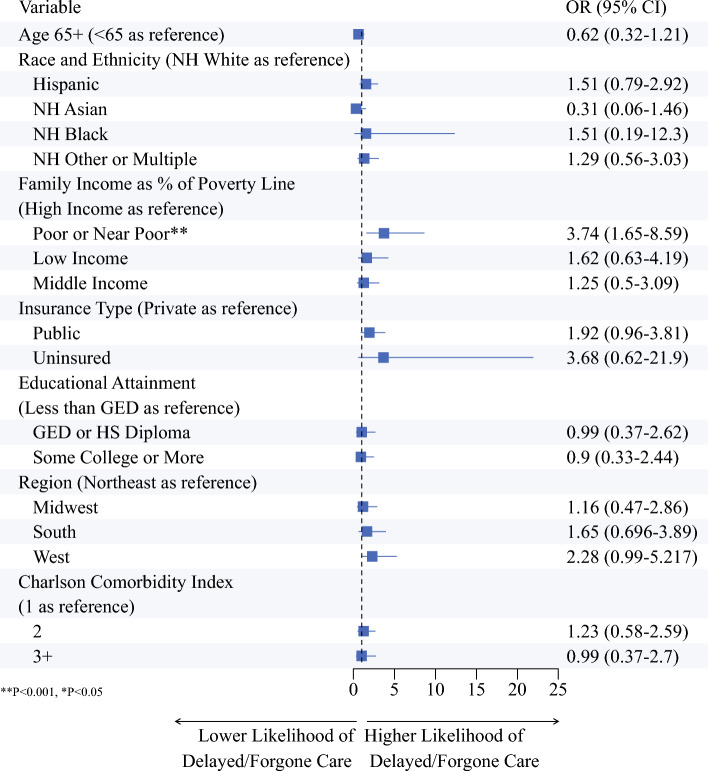
Associations between sociodemographic variables and delayed/forgone medical care in univariate logistic regression models. Odds ratios displayed are unadjusted. *NH* non-Hispanic; *GED* general educational diploma; *HS* high school

**Fig. 3:**
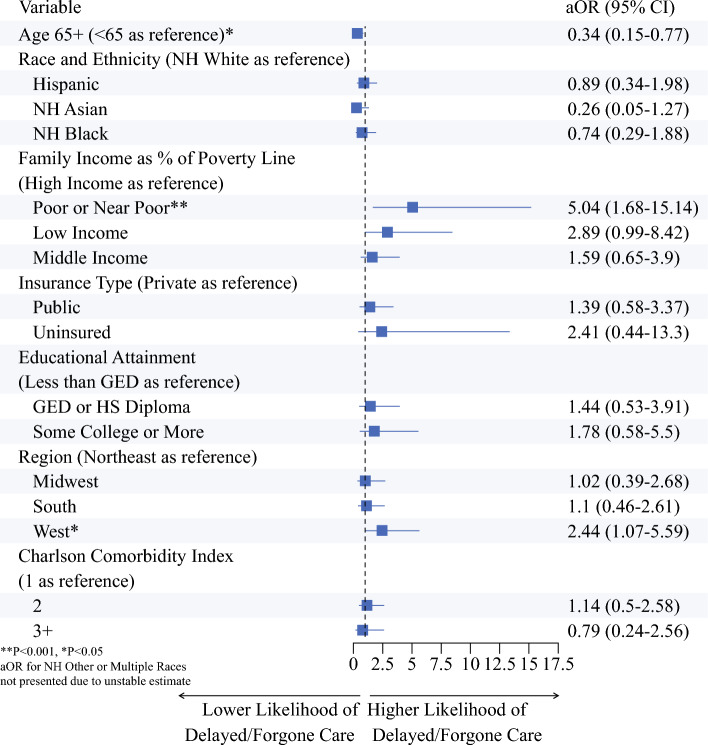
Associations between sociodemographic variables and delayed/forgone medical care in multivariate adjusted logistic regression model. Odds ratios displayed are fully adjusted. *NH* non-Hispanic; *GED* general educational diploma; *HS* high school

In univariate logistic regression models estimating probability of D/F care, poor or near poor family income was associated with increased odds of D/F care (odds ratio [OR] 3.74, 95% confidence interval [CI] 1.65–8.59, *P* < 0.001) (Fig. 2). In the multivariate adjusted logistic regression model, age ≥ 65 years was associated with decreased odds of D/F care (adjusted odds ratio [aOR] 0.34, 95% CI 0.15–0.77, *P* = 0.01). Living in the west (aOR 2.44, 95% CI 1.07–5.59, *P* = 0.03) and poor or near poor family income (aOR 5.04, 95% CI 1.68–15.14, *P* < 0.001) were associated with increased odds of D/F care (Fig. 3).

**Fig. 4:**
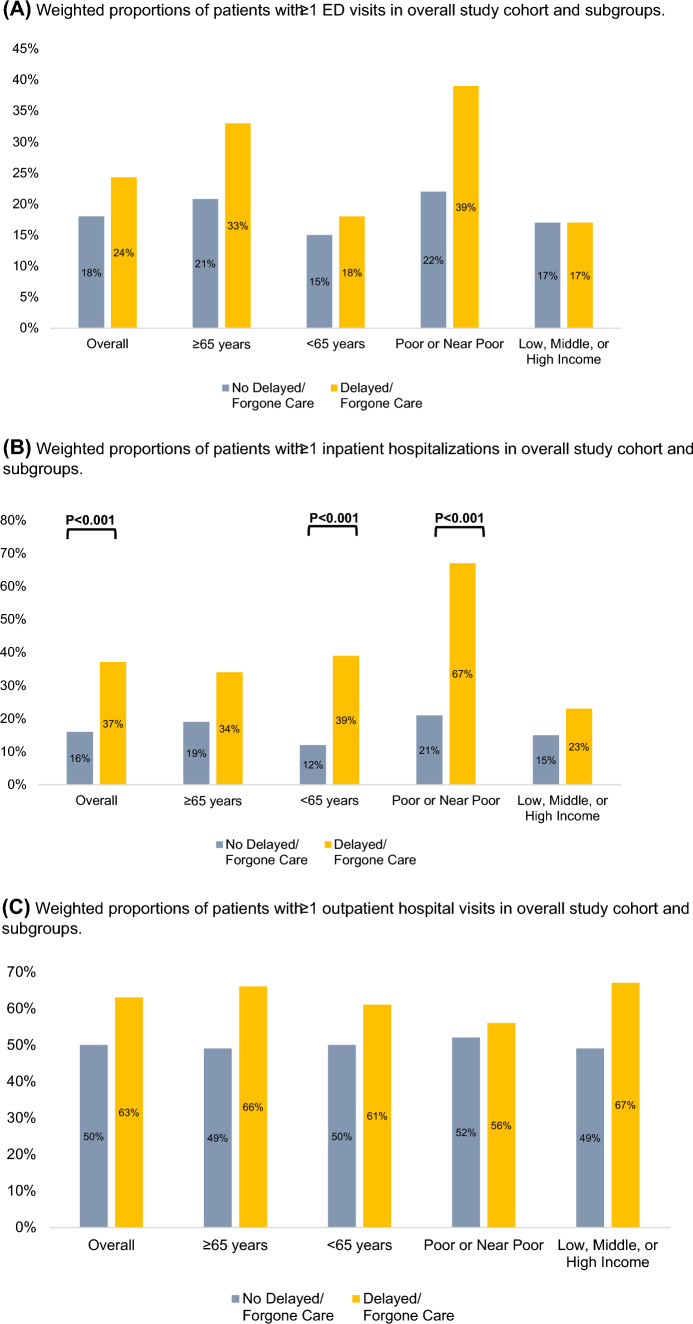

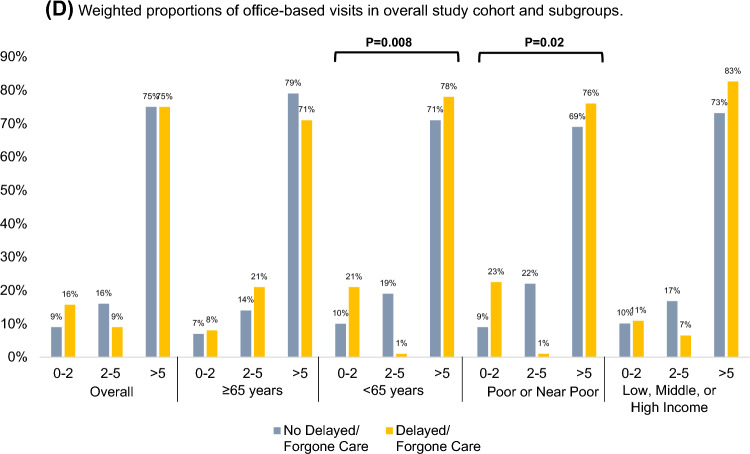
Differences in weighted proportions emergency department [ED], inpatient, outpatient, and office-based visits between patients reporting delayed/forgone medical care versus patients not reporting delayed/forgone medical care. **A** Weighted proportions of patients with ≥1 ED visits in overall study cohort and subgroups. **B** Weighted proportions of patients with ≥1 inpatient hospitalizations in overall study cohort and subgroups. **C** Weighted proportions of patients with ≥1 outpatient hospital visits in overall study cohort and subgroups. **D** Weighted proportions of office-based visits in overall study cohort and subgroups

The weighted proportion of patients with D/F care who had ≥ 1 ED visit in the prior year was 24%, which was nonsignificantly higher than the 18% of patients without D/F care who had ≥ 1 ED visit (Fig. 4A). A larger proportion of patients with D/F care had ≥ 1 hospitalization than patients without D/F care (37% vs. 16%, *P* < 0.001). Similar results were obtained in subgroups of patients < 65 years of age. Among patients whose family income was poor or near poor, 67% of patients with D/F care had ≥1 hospitalization compared to only 21% of patients without D/F care (*P* < 0.001) (Fig. 4B). There were no significant differences in the proportions of patients with ≥ 1 outpatient visits (Fig. 4C). Further, there were no significant differences in the proportions of patients experiencing > 5 office-based visits in the overall cohort. However, a higher proportion of patients reporting D/F care had > 5 office-based visits compared to patients not reporting D/F care in the < 65 years of age (78% vs. 71%, *P* = 0.008) and poor or near poor family income (76% vs. 69%, *P* = 0.02) subgroups (Fig. 4D).

Results of the two-part models of healthcare expenditures are summarized in Table 2. There were no significant differences in annual per capita total, out-of-pocket, ED, outpatient, office-based visit, or prescription medication expenditures between patients who experienced D/F care versus those who did not. However, patients experiencing D/F care had inpatient expenditures that were $5372 (95% CI $35–$10,709, *P* = 0.04) higher than patients who did not experience D/F.

**Table 2:** Results of two-part model of health care expenditures among adult women with breast cancer in MEPS, 2007–2017

	Difference in per capita annual expenditures for those with delayed/forgone care compared with those without delayed/forgone care	95% CI	*P*
Overall total expenditures, $	$6587	−$1357 to $14,530	0.1
Overall out-of-pocket expenditures, $	−$405	−$862 to $51	0.08
ED expenditures, $	$120	−$108 to $349	0.3
Inpatient expenditures, $	$5372	$35 to $10,709	0.04
Outpatient expenditures, $	$229	−$1675 to $2133	0.81
Office-based visit expenditures, $	$1057	−$2673 to $4786	0.56
Prescription medication expenditures, $	−$640	−$2036 to $757	0.37

All dollar values are presented as 2017 USD. All models were adjusted for insurance status (insured versus uninsured)

*CI* confidence interval; *ED* emergency department

Unweighted counts of reasons for D/F care reported by patients are summarized in Table 3. Overall, 32 patients reported financial reasons for D/F care, 43 patients reported nonfinancial reasons for D/F care, and 24 patients reported “Other.” We identified five patients in the cohort who experienced both delayed and forgone care and reported a distinct reason for each. One of those five patients reported a mixture of nonfinancial and financial reasons for D/F care. Among the remaining patients experiencing D/F care, 42.9% reported financial barriers as the primary reason for D/F care, 24.3% reported nonfinancial barriers as the primary reason for D/F care, and 31.4% of patients reported “Other” as the primary reason for D/F care.

**Table 3:** Self-reported reasons for delayed and/or forgone medical care among patients with breast cancer, 2007–2017

Reason for delayed/forgone care	Unweighted count of patients reporting reason for D/F care
Financial reasons	32
Could not afford care	20
Insurance company would not approve/cover/pay	11
Doctor refused family insurance plan	1
Nonfinancial reasons	43
Problems getting to doctor’s office	5
Different language	0
Could not get time off work	2
Do not know where to go for care	1
Was refused services	7
Could not get childcare	0
Did not have time or took too long	4
Other	24

All counts provided are unweighted

We performed sensitivity analyses in the unweighted sample to identify predictors of D/F care and differences in resource use and expenditures. Similar results to the weighted analyses were obtained with regards to predictors of D/F care, proportions of patients with ≥ 1 hospitalizations, and differences in per capita annual inpatient expenditures.

## Discussion

Approximately 5% of adult women with breast cancer in the United States reported experiencing D/F care. Among patients experiencing D/F care, 43% cited financial barriers and 24% reported nonfinancial barriers as the primary reason for D/F care. Among demographic factors associated with experiencing D/F care, poor or near poor family income and residing in the west correlated with significantly increased odds of D/F care. Age ≥ 65 years was associated with reduced odds of D/F care. Patients experiencing D/F care had significantly higher rates of inpatient hospitalizations than those who did not experience D/F care, and this gap was significantly larger in the subgroup of patients of poor or near poor family income. Additionally, patients experiencing D/F care had greater than $5000 greater annual, per capita inpatient expenditures relative to patients who did not experience D/F care. These findings suggest that reduced access to medical care leading to delays and/or forgone care may be a key driver of rising expenditures for cancer care. The findings of this study are consistent with previous findings that D/F care is associated with higher resource utilization and healthcare expenditures in other disease populations.^12–14^

We identified an increase in hospitalizations that coincided with an increase in per capita inpatient expenditures among individuals experiencing D/F care. It is possible, therefore, that increased inpatient expenditures may be primarily driven by an increase in the number of overall inpatient admissions among those experiencing D/F care. Increasing costs of cancer care have historically been driven by inpatient hospitalizations. However, with increasingly effective chemotherapies and pharmacotherapies provided in the ambulatory setting, outpatient, and prescription medication costs comprise a growing proportion of total cancer care costs.^15^ In this study, we found no significant differences in out-of-pocket, ED, outpatient, office-based or prescription medication expenditures between those reporting D/F care and those who did not. Nonetheless, previous work has found that ambulatory care costs account for greater than 30% of healthcare spending for breast cancer.^16^ Additionally, while ED expenditures comprise a small proportion of overall breast cancer care costs, there is evidence that potentially preventable ED visits are more common among breast cancer patients than patients with other types of cancer.^16,17^ These preventable visits may introduce significant unplanned cost burdens on breast cancer patients. Furthermore, the breakdown of healthcare expenditures among patients with breast cancer varies with insurance type, age, and tumor stage.^7,18–20^ Thus, the increase in inpatient resource use observed in this study warrants further investigation. Focused efforts to reduce D/F care as a means to reducing inpatient expenditures can have significant impacts on cost burdens faced by hospital systems caring for patients with breast cancer.

Differences in hospitalization rates between those experiencing D/F care and those who did not were similar among individuals aged < 65 years. Because being uninsured is highly prevalent among individuals < 65 years, insurance coverage may be a driving factor of increased rates of D/F care and resource utilization among nonelderly patients with breast cancer.^21^ However, uninsurance rates among women < 65 years vary by age: 10% of women 40–49 years, 8% of women 50–59 years, and 6.8% of women 60–64 years do not have any health insurance coverage according to the 2022 American Community Survey sample.^22^ Prior work has found that younger women with breast cancer face longer diagnostic delays than older women and that symptomatic presentation was correlated with delays.^23,24^ Because breast cancer in younger women tends to present with more aggressive disease that requires concomitantly aggressive treatment, delays in care could have greater implications for resource use, which was observed in the higher rate of inpatient hospitalizations in the < 65 years group.^25^ Moreover, younger women of low socioeconomic status (SES) with breast cancer are more likely to delay medical care than young women of high SES.^26^

We also found a notable difference in hospitalization rates by D/F status among poor or near poor patients. This finding aligns with our data on reasons for D/F care; 42.9% of patients experienced D/F care citing financial factors as the primary barrier for D/F care. However, we underscore the fact that the causal pathways for delayed care are multifactorial. Insurance coverage and affordability are just two of many factors that may motivate patients of low SES to delay care. Prior work has demonstrated that psychological barriers (e.g., fear), physical ailments (e.g., pain), and practical challenges (e.g., limited access to transportation) are all considerations in delayed care among patients of low SES,^27,28^ as well as patients from racial and ethnic minority groups. Additional work is needed to better address the specific barriers faced by historically marginalized patients in accessing timely breast cancer care.^29^

This study has several limitations. First, the cross-sectional nature of this study precludes causal inference regarding the relationship between D/F care and resource utilization. Second, owing to the small sample size of patients included in the study cohort, granular subanalyses of the specific reasons for D/F care and the type of care that is delayed and/or foregone by each patient could not be adequately powered. Third, as MEPS data is provided in the form of self-reported surveys, there is a possibility of recall bias among survey respondents. Fourth, prior studies analyzing MEPS data have underestimated disease prevalence, healthcare expenditures, and measures of resource utilization, so the estimates obtained in our study may be similarly deflated.^12,30^ Fifth, we presented unweighted counts of the primary reasons patients cited in MEPS for experiencing D/F care, and patients were limited to choosing only one reason for which they experienced delayed and/or foregone care. However, further analyses and data are needed to precisely determine the causal mechanisms by which D/F care arises, because it is likely that social circumstances including SES, insurance coverage, and negative experiences with the healthcare system as well as clinical factors, including comorbidities and lack of primary care access, interact in complex ways to give rise to D/F care.^28,31–33^

## Conclusions

Approximately 5% of adult women with breast cancer in the United States experience D/F care, and 43% cite financial barriers as the primary reason. Delayed/forgone care was associated with increased hospitalizations among all patients, particularly those < 65 years and who were poor. Delayed/forgone care was also associated with significantly greater inpatient expenditures. Future research should identify the causal mechanisms underlying D/F care to forestall the rising costs associated with nonreceipt of breast cancer care.

## Supplementary Information

Below is the link to the electronic supplementary material.

Supplementary file1 (DOCX 14 kb)

## Data Availability

All data used in this study are publicly available from the Medical Expenditure Panel Survey (MEPS) of the Agency of Healthcare Research and Quality.
