# Supplementary material for: Association Between Delayed/Forgone Medical Care and Resource Utilization Among Women with Breast Cancer in the United States
Source: Ann Surg Oncol. 2024 Dec 18;32(4):2534–44. doi: 10.1245/s10434-024-16586-x (PMC11882630; doi:10.1245/s10434-024-16586-x)
Supplement: Supplementary file 1 — Supplementary file1 (DOCX 14 kb) [file 10434_2024_16586_MOESM1_ESM.docx]

Reddy KP, et al. Association between delayed/forgone medical care and resource utilization among women with breast cancer in the United States

**Supplemental Material**

**Supplemental Methods 1**. Mapping of reasons for D/F care in MEPS to three mutually exclusive bins.

| **Reason Provided in MEPS** | **Categorization** |
| --- | --- |
| Could not afford care | Financial |
| Insurance company would not approve/cover/pay | Financial |
| Doctor refused family insurance plan | Financial |
| Problems getting to doctor’s office | Non-Financial |
| Language barriers | Non-Financial |
| Could not get time off work | Non-Financial |
| Don’t know where to go to get care | Non-Financial |
| Was refused services | Non-Financial |
| Could not get childcare | Non-Financial |
| Did not have time or took too long | Non-Financial |
| Other | Other |
